# A Large-Range and High-Sensitivity Fiber-Optic Fabry–Perot Pressure Sensor Based on a Membrane-Hole-Base Structure

**DOI:** 10.3390/mi15020174

**Published:** 2024-01-24

**Authors:** Bowen Duan, Zhenyin Hai, Maocheng Guo, Yongqiu Zheng, Jiamin Chen, Jiandong Bai, Zhixuan Su, Rui Liang, Hongtian Zhu, Qi Zhang, Chenyang Xue

**Affiliations:** 1 School of Aerospace Engineering, Xiamen University, Xiamen 361102, China; 19920211151508@stu.xmu.edu.cn (B.D.); 19920221151622@stu.xmu.edu.cn (M.G.); 19920221151557@stu.xmu.edu.cn (Z.S.); 35020230156555@stu.xmu.edu.cn (H.Z.); 19920231151668@stu.xmu.edu.cn (Q.Z.); 2 Stale Key Laboratory of Dynamic Measurement Technology, North University of China, Taiyuan 030051, China; zhengyongqiu@nuc.edu.cn (Y.Z.); 18335160365@163.com (J.C.); jdbai@nuc.edu.cn (J.B.); s202206095@st.nuc.edu.cn (R.L.)

**Keywords:** fiber-optic sensors, high-temperature pressure measurement, Fabry–Perot cavity, high sensitivity, wide pressure range

## Abstract

In the field of in situ measurement of high-temperature pressure, fiber-optic Fabry–Perot pressure sensors have been extensively studied and applied in recent years thanks to their compact size and excellent anti-interference and anti-shock capabilities. However, such sensors have high technological difficulty, limited pressure measurement range, and low sensitivity. This paper proposes a fiber-optic Fabry–Perot pressure sensor based on a membrane-hole-base structure. The sensitive core was fabricated by laser cutting technology and direct bonding technology of three-layer sapphire and develops a supporting large-cavity-length demodulation algorithm for the sensor’s Fabry–Perot cavity. The sensor exhibits enhanced sensitivity, a simplified structure, convenient preparation procedures, as well as improved pressure resistance and anti-harsh environment capabilities, and has large-range pressure sensing capability of 0–10 MPa in the temperature range of 20–370 °C. The sensor sensitivity is 918.9 nm/MPa, the temperature coefficient is 0.0695 nm/(MPa∙°C), and the error over the full temperature range is better than 2.312%.

## 1. Introduction

In high-temperature and high-pressure environments, such as those found in aviation engines, oil wells underground operations, and the nuclear industry, it is crucial to measure and monitor environmental pressure in real time and with precision [1,2,3]. In such extreme and challenging environments, commonly used resistive and piezoelectric sensors often experience failure [4,5,6]. The operational temperature of traditional silicon-based pressure sensors is limited to 250 °C. Although applying SOI material to pressure sensors is an attempt to surpass this limitation, achieving a long-term stable operating temperature beyond 500 °C remains challenging [7,8,9,10]. Moreover, pressure sensor chips made from SiC material have become a hot research topic. As a high-temperature piezoresistive material, SiC exhibits excellent electrical and mechanical properties. However, at temperatures exceeding 600 °C, the electrical characteristics of SiC noticeably degrade, and the introduced electrode materials in the sensor can restrict its overall performance [11,12,13]. The development and application of Fabry–Perot cavity optical pressure sensors have emerged to meet the demands of such extreme conditions. They possess characteristics such as high stability, high-temperature resistance, and high accuracy, making measurements in ultra-high-temperature environments more accessible and precise. Common Fabry–Perot cavity optical pressure sensors are primarily divided into diaphragm-type and fiber-in-cavity-type. In comparison to the diaphragm-type, the fiber-in-cavity-type has advantages of compact size and uniform material but faces challenges in working under large pressure ranges and exhibits lower sensitivity. It is more susceptible to interference in high-temperature environments. Therefore, opting for high-melting-point materials to fabricate diaphragm-type pressure sensors can effectively enhance the pressure working range of high-temperature pressure sensors [14,15,16,17,18,19]. By comparing the performance of the sensors in the studies, it becomes evident that the pressure range in most research is less than 1 MPa. This limitation falls short of meeting the pressure range requirements for harsh environmental conditions. Additionally, the relatively low sensitivity of the sensors exacerbates the impact of external factors, such as temperature drift, on sensor performance.

Sapphire (α-Al_2_O_3_), with a melting point of 2040 °C, possesses excellent optical properties and insulation, making it an ideal material for high-temperature sensors. A fully sapphire pressure sensor has the potential to achieve precise pressure measurements at temperatures exceeding 1500 °C [20]. In 2019, Wangwang Li proposed a high-temperature pressure sensor based on sapphire direct bonding, utilizing bonding technology to fabricate a sealed pressure-sensitive core with a pressure range of 700 kPa [21]. In 2020, Z. Wang presented a non-adhesive encapsulation sapphire Fabry–Perot interferometer, featuring zirconia sleeves and a zirconia shell to enhance performance. This sensor exhibits outstanding applicability, with a temperature measurement range of −50 °C to 1200 °C and a pressure measurement range of 0.4 to 4.0 MPa. Remarkable pressure sensitivity of 1.20 nm/MPa was observed at a high temperature of 1200 °C [22]. In 2021, Jihaeng Yi introduced a Fabry–Perot pressure sensor formed by ion-etching sapphire and directly bonding dual-layer chips, offering a pressure measurement range of 2.1 MPa and a sensitivity of approximately 350 nm/MPa [23]. In 2022, Suwei Wang introduced a MEMS-based fiber-optic Fabry–Perot high-temperature pressure sensor. The reflectance of the Fabry cavity was enhanced through coating technology. It operates within a temperature range of 25–300 °C, offering a pressure range of 0.5 MPa, with a corresponding sensitivity of 55.468 nm/MPa [24]. Z.Q. Shao proposed a full-sapphire Fabry–Perot pressure sensor based on wet etching and direct bonding, with a pressure range up to 5 MPa and a sensitivity of 548.1 nm/MPa. However, the production process is complex and time consuming, and the high-temperature performance has not been verified [25]. Oxsensis’ optical dynamic sensor, utilizing a sapphire diaphragm core, is capable of prolonged pressure measurements at 600 degrees Celsius, achieving a pressure of 4.5 MPa. Existing research on Fabry–Perot cavities designed for high-temperature environments typically employs materials such as quartz and sapphire. These cavities are formed by etching and bonding wafers, resulting in the creation of miniature Fabry–Perot structures. However, microcavity structures exhibit drawbacks, such as limited pressure ranges and low sensitivity. Some microcavity structures face challenges due to high roughness resulting from etching, necessitating coating for efficient reflection of optical signals. It is noteworthy that the choice of coating material can reduce the operational temperature of the sensor. Upon scrutinizing the performance of sensors in the aforementioned studies, it is evident that most research yields pressure ranges below 1 MPa. This falls short of meeting the pressure range requirements for harsh environmental conditions. Additionally, the relatively low sensitivity of these sensors amplifies the impact of external factors, such as temperature drift, on sensor performance.

To address the problem of pressure measurement in high-temperature and high-pressure environments, this article proposes a fiber-optic Fabry–Perot pressure sensor based on a membrane-hole-base structure; the use of a large-cavity structure significantly increases the sensor’s pressure range and sensitivity. It utilizes a three-layer direct bonding structure made of sapphire, which is simple in design, high in strength, and can be efficiently produced in batches. This design avoids the issue of different thermal strain coefficients caused by etching and coating processes, improving the sensor’s temperature resistance and thermal stability. In addition, the structure also results in a significant increase in the Fabry–Perot cavity length, which is not matched by the existing micro-cavity demodulation system. To address this issue, a supporting large-cavity demodulation system was developed, successfully solving the demodulation problem for Fabry–Perot cavities with a diameter of 200 μm and a pressure range of 0 to 10 MPa.

## 2. Sensor’s Structure and Principle

**Figure 1:**
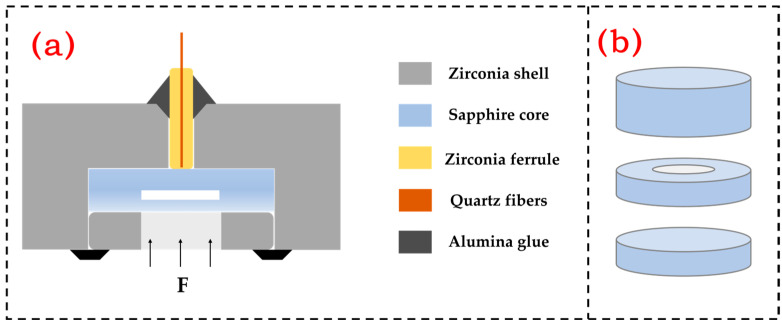
(**a**) Schematic diagram of the pressure sensor structure; (**b**) Schematic diagram of the sensitive core structure.

Figure 1a is a schematic diagram of the structure of the fiber-optic Fabry–Perot pressure sensor, which is mainly composed of an all-sapphire Fabry–Perot cavity, a quartz optical fiber, a zirconia ferrule, a zirconia sleeve, and an alumina high-temperature glue. Among them, the sapphire Fabry–Perot cavity is composed of three layers of structure—the schematic diagram of the structure is shown in Figure 1b—which are the pressure diaphragm under pressure, the air cavity diaphragm that forms the cavity structure, and the basement diaphragm close to the optical fiber, and the three diaphragms form the cavity structure through direct bonding.

When pressure is applied to the fiber-optic Fabry–Perot pressure sensor, the wafer will deform and directly translate into a change in the length of the Fabry–Perot cavity. The deformation of the diaphragm is calculated based on the theory of small deflection deformation in elastic mechanics, which states that the deformation of the center of the sensitive silicon diaphragm under load can be calculated by the following equation:

(1) $$\frac{Pr^{4}}{Eh^{4}}=\frac{16}{3\left(1\;-u^{2}\right)}\frac{y}{h}$$

In the formula, P is the pressure applied to the diaphragm; r is the radius of the effective pressure position of the diaphragm; E is the Young’s modulus of the diaphragm material; h is the thickness of the circular diaphragm; y is the deformation of the diaphragm; and u is the Poisson ratio of the diaphragm. It can be seen from the formula that the deformation of the diaphragm, that is, the change of the air cavity, is linearly related to the pressure.

**Figure 2:**
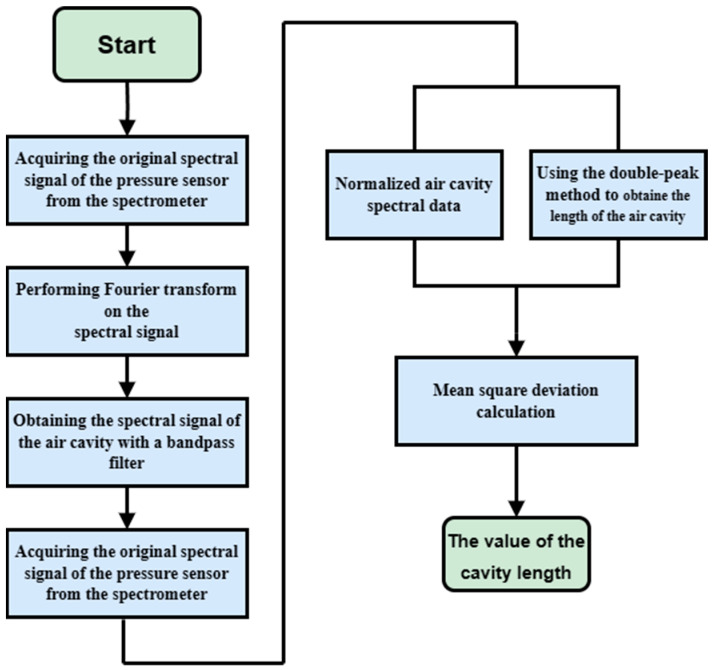
Demodulation system flow chart.

**Figure 3:**
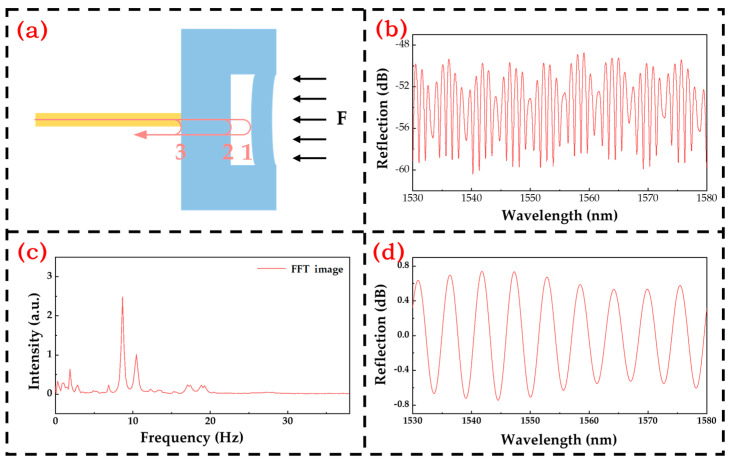
(**a**) Optical path diagram; (**b**) Reflection spectra at normal temperature and pressure; (**c**) The image obtained by Fourier transform of the spectrum; (**d**) Air cavity spectra obtained by Fourier inversion.

The demodulation system’s flowchart is depicted in Figure 2. As light penetrates the sensor through the optical fiber, it undergoes reflection and transmission at each reflective surface within the sensor core, as illustrated in Figure 3a. The resulting reflected light beams intersect and superimpose, giving rise to a reflection spectrum, as displayed in Figure 3b. Upon Fourier transformation of the spectrum, distinct peaks emerge, as depicted in Figure 3c. The leftmost peak corresponds to the air cavity, the second peak to the substrate, and the third peak results from the superposition of the air cavity and substrate. The target air cavity peak is isolated, followed by an inverse Fourier transform, as visualized in Figure 3d. Post normalization, the length of the Fabry–Perot cavity is computed utilizing the mean square deviation method based on the double-peak method’s cavity length calculation. Consequently, the variation in cavity length is determined to ascertain the pressure value [26,27,28].

To miniaturize the sensor, the pressure diaphragm and air cavity diaphragm use double-polished c-axis sapphire wafers with the minimum thickness, which is approximately 216 μm. To make the spectral image peak distinguishable, a 600 μm double-polished sapphire wafer is selected as the base. The sensor diaphragm should ensure that the sensitive diaphragm is within the elastic deformation range. According to the linear principle, that is, the maximum deformation of the pressure-sensitive diaphragm does not exceed 1/5 of the diaphragm thickness, then

(2) $$y_{\max}=\frac{3\left(1-\mu^{2}\right)Pr^{4}}{16Eh^{3}}<0.2\;h$$

With the parameters considered, the maximum radius of the pressure diaphragm is calculated to be 2.9 mm at a pressure range of 10 MPa. Considering the connection method between the core and the optical fiber, the radius of the inner hole of the Fabry–Perot cavity is selected to be 2 mm, and the overall radius is 4 mm. The parameters are shown in Table 1.

## 3. Fabrication of Sensor

**Figure 4:**
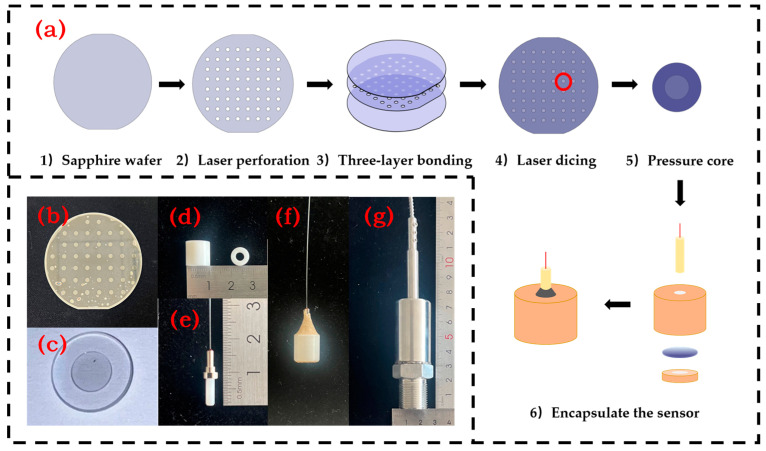
(**a**) Pressure core system process; (**b**) Annealed sapphire three-layer diaphragm; (**c**) The cut sapphire pressure core; (**d**) Zirconia bushing; (**e**) Zirconia fiber core; (**f**) Pressure sensor after high-temperature glue fixing; (**g**) Packaged pressure sensor.

The manufacturing process of the fiber-optic Fabry–Perot pressure sensor is shown in Figure 4a. A 4-inch double-polished sapphire wafer is selected as the core material, with a surface roughness of 0.02 μm. Firstly, a laser (wavelength of 355 nm, frequency of 700 kHz, power of 14.3 W) is used to drill and cut the wafer, creating an air cavity for the core, with a hole radius of 2 mm. Then, the sapphire wafer is cleaned and oxygen plasma activated for 20 min. It is then placed in the bonding machine (SSUS, XB8, Germany) in sequence and pre-bonded with a pressure of 25,000 N and a temperature of 450 °C for 4 h. After removal, it is annealed in a high-temperature furnace at 1100 °C for 2 h. Then, the three layers of bonded wafers are cut with a hole radius of 4 mm to form a fully sapphire Fabry–Perot cavity. Figure 4b shows the three layers of sapphire wafers after annealing. Figure 4c shows the pressure core after cutting.

Subsequently, using a zirconia sleeve (customized, outer diameter 5 mm, height 10 mm, inner diameter 4 mm), high-temperature-resistant inorganic glue (Spring Polymer XY803, China), and a zirconia-inserted fiber-optic insert (customized, outer diameter 2.5 mm), the fiber optic was fixed perpendicularly to the pressure core. The zirconia sleeve structure is shown in Figure 4d, and the physical diagram of the insert is shown in Figure 4e. The pressure core was inserted into the zirconia sleeve, covered with a press plate to fix the pressure core, and fixed with high-temperature glue. After that, the insert was inserted and adjusted, and then fixed with high-temperature glue at the connection between the insert and the zirconia sleeve, as shown in Figure 4f. To fix the sensor in a high-temperature and high-pressure furnace, a 304 stainless-steel (customized, M20) encapsulation was used. The sensor was placed in the stainless-steel encapsulation and sealed with high-temperature glue to prevent leakage. The finished product is shown in Figure 4g.

## 4. Characteristics of the Sensor

### 4.1. Test of Pressure and Temperature Resistance of Sensors

**Figure 5:**
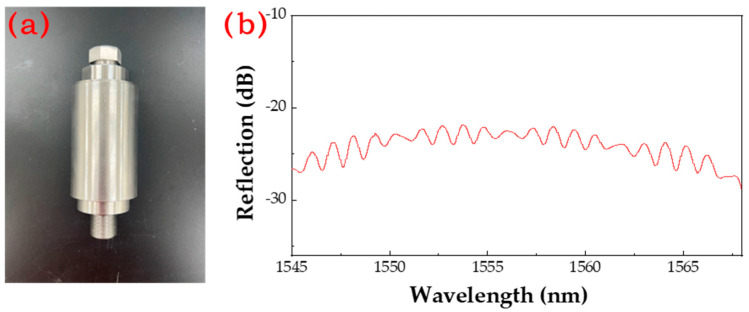
(**a**) Pressure tanks; (**b**) Spectral signal of the sensor at normal pressure and 700 °C.

To verify the pressure resistance performance of the sensor, the pressure-sensitive core of the sensor was placed in dye oil and pressurized. Absence of dye oil in the core indicated that the core had no leakage. A pressure tank (customized, Const), as shown in Figure 5a, was employed. Three sensitive cores and dye oil were placed in the pressure tank under a normal-temperature environment and configured on a liquid pressurizer for pressurization. The pressure plate was weighted to 10 MPa for 10 min, and there was no dye oil in the cavity after removal. The results showed that the upper pressure limit of the pressure-sensitive core was higher than 10 MPa.

The sapphire-bonded core, operating under normal atmospheric pressure, exhibits a temperature ceiling for signal output surpassing 700 °C. In a high-temperature environment of 700 °C, the signal demonstrates sustained output for over 30 min. However, over time, owing to the inherent disparities between the pressure sensor core and the optical fiber material, the optical signal’s contrast gradually diminishes, resulting in a weakened output signal spectrum, as illustrated in Figure 5b. Constrained by the inadequate demodulation capability for low-contrast signals in high-temperature states, the stable demodulation of the Fabry–Perot cavity length becomes challenging. Consequently, continuous full-scale testing at 700 °C proves unattainable. Through experimental testing, it is established that the highest temperature achievable for stable demodulation is 370 °C; subsequent substitution of sapphire optical fiber for quartz optical fiber would effectively address this issue.

### 4.2. Sensor Performance Test

**Figure 6:**
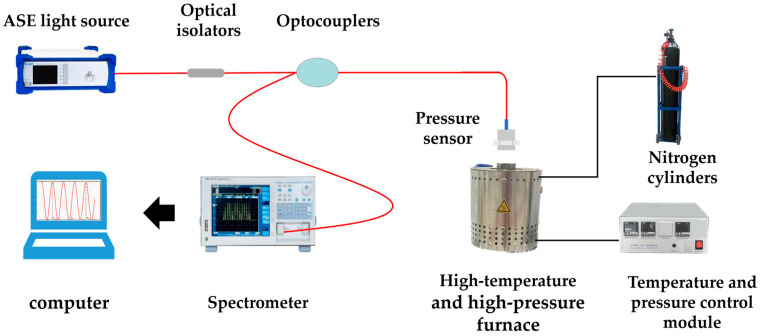
High-temperature and high-pressure test platform.

As shown in Figure 6, a high-temperature and high-pressure testing platform was built, including a fiber-optic Fabry–Perot pressure sensor testing system and a temperature and pressure control system. The fiber-optic Fabry–Perot pressure sensor sensing system consists of a pressure sensor, ASE light source (Golight, China), spectrograph (Yokogawa, AQ6370D, Japan), coupler, and computer. The pressure control system is composed of a high-temperature and high-pressure furnace, nitrogen cylinder, temperature controller, digital pressure indicator, and digital temperature indicator. The temperature range of the system is 10–700 °C, the pressure range is 0–10 MPa, the temperature control accuracy is ±2 °C, and the pressure control accuracy is 0.5%F.S. The sensor is connected to the high-temperature and high-pressure furnace through a threaded connection in a stainless-steel package, with a thermocouple and pressure gauge at the connection point for numerical calibration and a digital display. The fiber-optic sensor is connected to the ASE light source and spectrograph through a fiber coupler.

**Figure 7:**
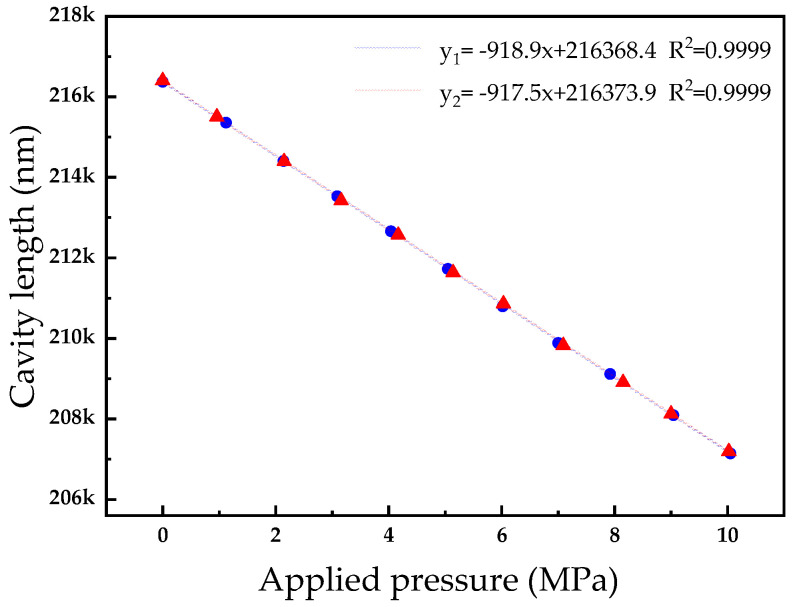
Contrast pressure–cavity length fitted lines.

To verify the repeatability of the sensor at room temperature, the pressure of the sensor was increased from 0 MPa to 10 MPa in steps of 1 MPa. The sensor spectrum was transmitted to the host computer through a spectrometer for demodulation, and the sensor Fabry–Perot cavity length was obtained. The pressure point corresponding to the cavity length was recorded. The process was repeated after 2 days, and the two-cavity length–pressure curves measured are shown in Figure 7. The test results show that the demodulation algorithm of the sensor is applicable, and the sensor has good repeatability; the Fabry–Perot cavity linearly shortens within the pressure range of 0–10 MPa, with a linearity of higher than 0.9999.

Subsequently, the pressure response under high-temperature conditions was studied. The pressure response of the sensor was tested at 20 °C, 90 °C, 160 °C, 230 °C, 300 °C, and 370 °C, and the pressure was stepped up to 10 MPa at each stage with the step length of 1 MPa, and the amount of change in the length of the air cavity obtained by demodulation was recorded.

**Figure 8:**
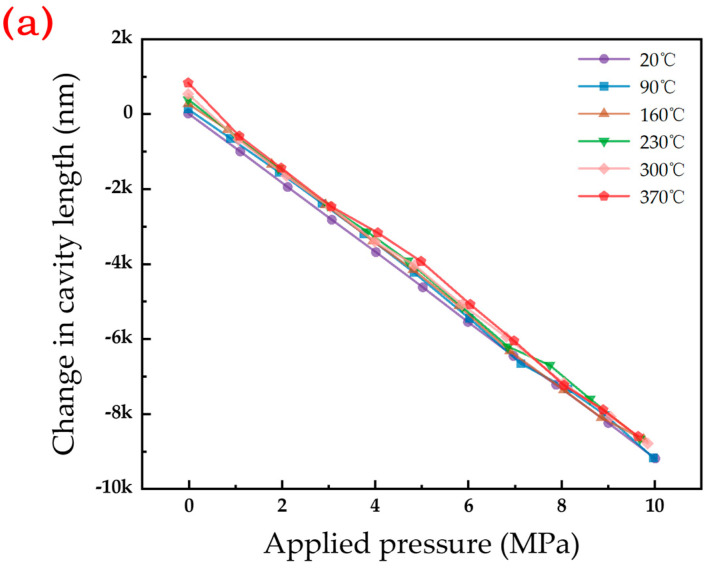

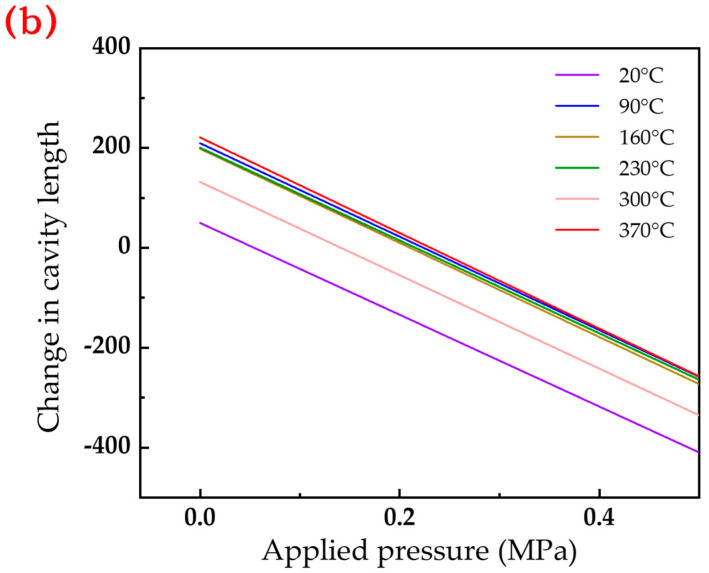
(**a**) The relationship between the applied pressure and the change in cavity length at different temperatures; (**b**) Partial enlarged fitted line.

**Figure 9:**
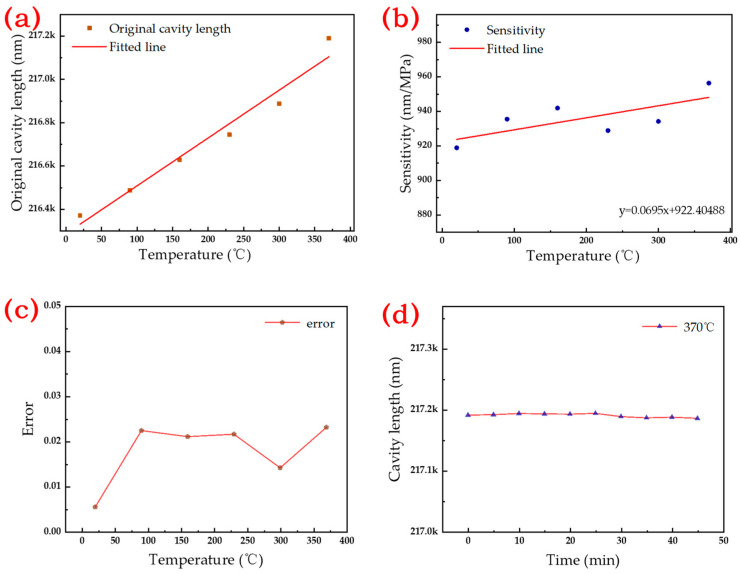
(**a**) Original cavity length at different temperatures; (**b**) Sensitivity at different temperatures; (**c**) Error at different temperatures; (**d**) Stability experiment of the sensor.

Figure 8a shows the relationship between cavity length and pressure between 20 °C and 370 °C; Figure 8b shows the local fitted line. The results show that the length of the Fabry–Perot cavity decreases with the increase in pressure. At temperatures of 20 °C, 90 °C, 160 °C, 230 °C, 300 °C, and 370 °C, Figure 9a,b illustrate the original cavity length and sensitivity of the sensor. The original cavity length values are recorded as 216,370.8, 216,487.1, 216,628.8, 216,744.8, 216,888.4, and 217,190.1 nm, respectively. Simultaneously, the pressure sensitivity is documented as 918.9, 935.5, 941.9, 928.9, 934.2, and 956.3 nm/MPa, respectively, for the corresponding temperature conditions. The error under different test temperatures is shown in Figure 9c; the original cavity length and sensitivity gradually increase as the temperature increases. In the measurement of the original cavity length data, the first five temperature points exhibit a relatively linear ascending trend. However, at 370 °C, the variation in the original cavity length is significantly higher than the preceding temperature points. The potential cause for this result might be attributed to the lack of precise temperature control in the testing environment. The high-temperature, high-pressure furnace, upon reaching the preset high temperature, pauses the heating process, resulting in a decrease in furnace temperature. When the temperature control module identifies this process after a certain delay, it resumes the heating. The limited space within the furnace, coupled with the substantial temperature changes during this process, leads to a discrepancy between the actual sensor temperature and the indicated temperature. In testing, the sensor, being closer to the heat source, experiences an actual temperature higher than 370 °C, thus causing a more significant increase in the original cavity length. At 230 and 300 °C, the sensitivity decreases, possibly due to the rapid heating rate, which exerts uneven stress on the material, which affects the deformation of the diaphragm. The error at different test temperatures is shown in Figure 9c, where the maximum nonlinearity range of the sensor is 2.312% over 10 MPa.

Finally, the thermal stability of the sensor was verified, and the air cavity length was measured every 3 min at 370 °C at atmospheric pressure for 45 min, as shown in Figure 9d. The length of the air cavity is stable at 216,760.6 nm, and the maximum variation is 4.693 nm. Based on the sensor sensitivity, this perturbation corresponds to a pressure change of 4.994 kPa, with a relative resolution of 0.49. These small changes may be due to unstable spectral signals and residual stress relief from the sensor. As a result, the manufactured sensors have good thermal stability and can be used for pressure measurement.

## 5. Conclusions

In this paper, a fiber-optic Fabry–Perot high-temperature pressure sensor for extreme high-temperature and high-pressure environments is proposed and manufactured, and a demodulation system is developed for the large-cavity length of the sensor. The sensitive core of the pressure sensor is made with sapphire three-layer bonding technology, which improves the pressure range and sensitivity of this kind of sensor. The experimental results show that the pressure range of the sensor is 0–10 MPa, the output signal temperature is 700 °C, and the demodulation temperature range is 370 °C. At room temperature, the pressure sensitivity registers at 918.9 nm/MPa, and the temperature coefficient within the range of 20–370 °C is determined to be 0.0695 nm/(MPa∙°C). The sensor exhibits an error in the full scale better than 2.312%, with a resolution of 0.499%F.S. The fiber-optic Fabry–Perot pressure sensor boasts notable attributes, including a broad pressure range, heightened sensitivity, straightforward structure, robust construction, and scalability for mass production. Its promising application prospects lie in the domain of high-temperature pressure testing. Subsequent research endeavors will concentrate on enhancing the demodulation capability, particularly for low-contrast spectral signals.

## Figures and Tables

**Table 1:** Sapphire pressure core parameters.

Parameters	Symbol	Units	Value
Thickness of diaphragm	h	μm	200
Young’s modulus of sensitive diaphragm	E	GPa	380
Poisson’s ratio of sensitive diaphragm	μ		0.27
Pressure range	P	MPa	8
Initial length of Fabry–Perot cavity	L	μm	200

## Data Availability

Data are contained within the article.
